# Generalization of beneficial exposure effects to untreated stimuli from another fear category

**DOI:** 10.1038/s41398-023-02698-7

**Published:** 2023-12-19

**Authors:** Iris Kodzaga, Ekrem Dere, Armin Zlomuzica

**Affiliations:** 1 Department of Behavioral and Clinical Neuroscience, Ruhr-University Bochum (RUB), D-44787 Bochum, Germany; 2 Sorbonne Université. UFR des Sciences de la Vie, F-75005 Paris, France

**Keywords:** Psychiatric disorders, Human behaviour

## Abstract

Previous research has shown that fear associated with one stimulus often spreads to other stimuli with similar perceptual features as well as across different stimulus categories. Exposure is considered as the most effective intervention to attenuate exaggerated fear. The extent to which exposure treatment effects can generalize to fears not targeted during treatment remains elusive. Previous studies on possible generalization of beneficial effects of exposure used stimuli sharing the same stimulus category and/or stimuli having high perceptual similarity. The current study examined whether exposure treatment generalization can be achieved for untreated stimuli which do not share any perceptual resemblance and belong to a different fear category. An analogue sample of fifty participants with fear of spiders (animal-related fears) and heights (natural environment-related fears) was tested. Participants have been randomly assigned to either an exposure treatment (*n* = 24) or a control condition (*n* = 26). Exposure treatment was designed to only target participants’ fear of spiders, leaving their fear of heights untreated. Results demonstrated that the effects of exposure treatment generalized to fear of heights, as indicated by a reduction in behavioral avoidance, as well as self-reported acrophobia symptoms. The present study confutes the assumption that generalization of exposure effects to untreated fears is based on perceptual similarity. Clearly, further research is required to determine the decisive factors, in order to expand the generalization effect permanently to any given type of fear.

## Introduction

Individuals who display a certain fear often show an increased predisposition to develop another fear [1]. Fear responses elicited by a stimulus often spread to other stimuli that share similar features or belong to the same category - a phenomenon known as fear generalization [2]. Attempts aimed to understand and identify the nature of fear generalization is therefore of utmost importance for both basic and clinical research. In the same vein, increased research effort has been made to attenuate and prevent spreading of fears [3, 4].

The most effective intervention to attenuate exaggerated fears is exposure therapy, which is considered a first-line evidence-based treatment for phobia and anxiety disorders [5–8]. According to the inhibitory learning model [9, 10] the beneficial effects of exposure therapy are mediated by fear extinction. In line with this assumption, the original CS-US association learned during fear conditioning is not affected by the extinction process. Instead, novel secondary inhibitory learning about the CS-US relationship is initiated bearing the information that the CS no longer predicts the occurrence of the US. Explanations of the exposure effect are based on the violation of expectations with regard to the CS-US contingency, fear habituation, belief disconfirmation and/or other mechanisms [11]. Fear generalization, a pathological marker of anxiety, might be linked to exposure outcomes. A recent study found that pre-treatment markers of fear generalization, such as overgeneralized fear responses and disrupted inhibitory brain networks, were associated with poor responses to behavioral exposure therapy in spider-phobic patients [12].

While exposure can lead to reductions in fear and avoidance, observations of therapeutic effects of exposure have usually been restricted to the treated context [13]. Further, the few studies investigating generalization of exposure effects to untreated stimuli have so far been limited to stimuli sharing the same stimulus class and/or stimuli with high perceptual similarity [14–17]. As such, it has been demonstrated that exposure-induced fear reduction does in fact generalize to different specimens of the treated stimulus [16, 17]. Moreover, we recently demonstrated that a generalization of therapeutic effects during exposure is possible across stimuli which belong to the same category yet differ perceptually from each other. In this regard, we provided initial evidence that subjects with fear of spiders and cockroaches who underwent an exposure for one phobic stimulus (spiders), whereas the other phobic stimulus (cockroaches) was left untreated, exhibited attenuated fear to both spiders and cockroaches. Beneficial effects of exposure for treated (spiders) and untreated (cockroaches) stimuli were observable at the behavioral, subjective, and physiological level [15]. Perceptual similarities between treated and untreated stimuli and a set of shared cognitive distortions related to common animal fears [2, 18, 19] may account for the previous findings. However, findings are thus far limited to untreated stimuli sharing the same stimulus category and bearing some degree of perceptual feature overlap with the treated stimuli.

Building on this previous research, the current study was therefore designed to test whether the observed exposure treatment generalization can also be demonstrated for untreated stimuli which do not share any perceptual resemblance and belong to a different fear category. Given that fears and phobias related to spiders and heights are relatively common in the general population [20], fear of spiders and heights were considered to be appropriate candidates for examining exposure generalization across different stimulus categories (animal vs. natural environment). To this end, subjects with both, fear of spiders and heights received an exposure treatment for their spider fear, while leaving their fear of heights untreated. Generalization of treatment effects to the untreated fear was determined after exposure therapy and compared to a waiting control group receiving no exposure. Observable generalization effects were expected to occur at various fear response levels, including avoidance behavior, subjective fear, and self-reported symptoms.

## Materials and methods

The study was pre-registered in the Clinical Trials.gov registry (https://clinicaltrials.gov/ct2/show/NCT03907345). The pre-registration was submitted prior to data collection, and included our hypotheses, detailed methods, and procedures, as well as exclusion and inclusion criteria.

### Primary and secondary outcome measures

In order to weight our study results according to their clinical importance, we have defined primary and secondary outcome measures. Primary outcome measures included changes in Behavioral Approach Tests (BATs), subjective fear levels and heart rate measurements in response to both spiders and heights from before to after exposure. These measures are considered to be more important for the translation into a clinical context (since they indicate a change in avoidance behavior), than mere changes in spider- and height-fear related questionnaires, which were therefore regarded as secondary outcome measures.

However, technical difficulties during data collection limited the availability of heart rate data to a restricted number of participants, compromising statistical power and data reliability. Additionally, the physically demanding nature of the height BAT, requiring participants to climb the church tower’s stairs, turned out to be a confounding factor that compromised the heart rate measurement as a physiological indicator of fear. Due to these confounds we decided not to report heart rate data.

### Power analysis

Prior to conducting the study, we performed a power analysis using G*Power version 3.1.9.7. Taking into account the factors of group (exposure vs. control) and the two measurement time points (pre-assessment vs. post-assessment), we aimed to achieve a power of 0.985 with an α-error rate of 0.05. Based on a study addressing a similar research question [15], we estimated an effect size of f = 0.30 for a repeated measures analysis of variance (ANOVA) with a within-between interaction. Based on the power analysis results, a sample size of 50 individuals was determined to be sufficient to achieve the desired level of statistical power. This sample size was chosen to ensure that our study would be adequately powered to detect the effects of interest and yield reliable and meaningful results.

### Participants

An analogue sample of participants was recruited via the online recruitment platform of the Ruhr University Bochum. Only individuals (with an age range of 18–40 years), who reported both a fear of heights and a fear of spiders have been included in the study. Acute psychological conditions, current psychological or psychiatric treatment, chronic medication, drug or alcohol abuse, pregnancy, medical, and chronic physical illness constituted exclusion criteria. All participants indicated normal or corrected-to-normal vision. A total of 171 subjects were screened, out of which 60 subjects met the inclusion criteria for participation and showed up for the first appointment. Nine participants were excluded because they did not demonstrate sufficient fear of heights (i.e., indicated by a score of 13 on the Behavioral Approach Test at pre-assessment). Another participant was excluded due to incomplete assessment. Thus, our analytic sample included *N* = 50 participants who were either assigned to the exposure (i.e., received one session of exposure treatment for spider fear; *n* = 24) or control condition (i.e., received no exposure; *n* = 26). Relevant demographic characteristics are summarized in Table 1. The study obtained approval from the local ethics committee of the Ruhr University Bochum and was conducted in line with the Declaration of Helsinki. All participants provided written informed consent and received 7.5 university course credits or €75 in compensation for their participation.

**Table 1:** Demographic characteristics at pre-assessment.

Variable	Exposure group		Control group		*P*-value
	M	*(SD)*	M	*(SD)*	
Age (years)	24.54	(*4.53*)	25.46	(*7.45*)	0.604
Gender (female)	79.2%		84.6%		0.616
Greater fear reaction (spiders)	70.8%		65.4%		0.680
BDI-II (α = 0.884)	6.48	(*5.93*)	6.08	(*6.65*)	0.828
STAI-S (α = 0.914)	39.71	(*10.14*)	40.71	(*9.91*)	0.731
STAI-T (α = 0.919)	37.67	(*10.34*)	39.42	(*9.60*)	0.547
GSE (α = 0.874)	30.63	(*4.60*)	28.88	(*4.67*)	0.134

The Cronbach’s Alpha values for each scale in our sample are provided in parentheses next to the respective abbreviations.

*BDI-II* Beck’s Depression Inventory-II, *STAI* State-Trait Anxiety Inventory, *GSE* General Self-Efficacy Scale, *SD* Standard Deviations.

### Fear-eliciting stimuli and experimental setting

The fear-eliciting stimulus was a non-venomous house spider (Tegenaria domestica). Each session started and ended in the same room, in which participants completed the diagnostic interview at the first appointment and filled out questionnaires in each session. The spider BAT and exposure treatment took place in a separate room containing the spider, as well as additional items used for exposure treatment, such as thick gloves, latex gloves, pencils, and a small jar. A nearby church tower (72 m) served as the setting for the height BAT.

## Methods

A set of diagnostic tools and questionnaires was used to assess the existence and severity of fears and possible coexisting mental disorders in our sample. 1) The Mini-DIPS, a short diagnostic interview for mental disorders (Mini-DIPS [21]) which is based on DSM-IV criteria; 2) the Beck’s Depression Inventory-II (BDI-II [22]); 3) the State-Trait Anxiety Inventory (STAI [23]) to control for any pre-existing differences in depressive and anxiety symptoms across participants; 4) the general self-efficacy questionnaire (GSE [24]). Further, at each appointment, three different questionnaires were used to assess spider-related fear and beliefs. These comprised the 5) Fear of Spiders Questionnaire (FSQ [25]), an 18 item questionnaire rated on a 7-point Likert scale covering different levels of spider fear, such as physiological reactions, subjective evaluation, and avoidance behavior; 6) the 31-item Spider Phobia Questionnaire (SPQ [26]), on which items are rated dichotomously as either true or false; and 7) the Spider Phobia Beliefs Questionnaire (SBQ [27]), which consists of 48 items measuring dysfunctional beliefs on visual analogue scales ranging from 0 (=not at all) to 100 (=completely). Lastly, 8) the Anxiety subscale of the Acrophobia Questionnaire (AQ [28]) was used to capture the anxiety elicited in different height-relevant situations. On all of these questionnaires, a higher score indicated a higher level of fear and/or unrealistic beliefs. Reference values and/or clinical cut-off scores of the employed measures are provided for the FSQ [29], SBQ [30], SPQ [31], and AQ [32] in the description of Table 2.

**Table 2:** Primary and secondary outcome measures at pre- and post-assessment.

Variable	Exposure group		Control group		*P*-value
	M	*(SD)*	M	*(SD)*	
Primary outcome measures					
Spider BAT					
BAT score pre	5.29	*(1.81)*	4.88	*(2.05)*	0.461
BAT score post	9.21	*(2.04)*	5.54	*(2.01)*	<0.001^***^
SUDS score pre	55.83	(*18.81*)	58.65	(*20.23*)	0.613
SUDS score post					
Initial	20.00	*(15.40)*	51.73	*(19.85)*	<0.001^***^
Final	46.88	*(24.66)*	63.46	*(22.19)*	0.012^*^
Height BAT					
BAT score pre	7.46	*(2.34)*	7.58	*(2.28)*	0.857
BAT score post	9.83	*(2.62)*	8.81	*(2.15)*	0.136
SUDS score pre	68.54	(*17.85*)	68.85	(*16.14*)	0.950
SUDS score post					
Initial	48.88	*(20.39)*	53.00	*(20.97)*	0.489
Final	67.50	*(22.94)*	72.27	*(15.02)*	0.385
Secondary outcome measures					
FSQ					
Pre (α = 0.901)	65.91	(*21.72*)	70.85	(*17.08*)	0.379
Post (α = 0.968)	26.70	(*16.49*)	71.88	(*19.25*)	<0.001^***^
SBQ					
Pre (α = 0.943)	25.27	(*8.15*)	27.31	(*7.94*)	0.386
Post (α = 0.978)	11.65	(*7.45*)	26.74	(*8.27*)	<0.001^***^
SPQ					
Pre (α = 0.765)	19.71	(*4.58*)	20.58	(*4.54*)	0.522
Post (α = 0.837)	15.71	(*5.22*)	20.42	(*4.86*)	0.003^**^
AQ					
Pre (α = 0.839)	58.64	(*19.84*)	67.41	(*14.70*)	0.103
Post (α = 0.898)	47.00	(*17.10*)	64.32	(*21.38*)	0.005^**^

Internal consistency (Cronbach’s Alpha values) for each scale in our sample is provided in parentheses below the respective abbreviations for the pre- and post-assessment.

*BAT* Behavioral Approach Test, *SUDS* Subjective Units of Distress Scale, *FSQ* Fear of Spiders Questionnaire, high fear ≥55, *SBQ* Spider Beliefs Questionnaire, high fear ≥26, *SPQ* Spider Phobia Questionnaire, clinically significant symptom severity ≥20, *AQ* Acrophobia Questionnaire, high fear ≥45.

Significant between-group differences from independent T-tests, with ^***^P < 0.001, ^**^P < 0.01, *P < 0.05.

### Behavioral approach test

In vivo behavioral approach tests (BAT) were conducted prior to, and after exposure treatment in the exposure group, to assess treatment efficacy with respect to fear and avoidance. In the control group, not receiving exposure, two BATs were performed with an inter-test interval of 1 week in order to assess the effects of task repetition, passage of time, and the reliability of the measuring tool (the individual ranks of the participants within the group should not change significantly). BATs were measured in terms of approach distance to the fear-eliciting stimulus and in terms of subjective ratings of fear levels at the closest distance to the fear-eliciting stimulus reached by the participant.

### BAT for spiders

The BAT for spiders was adapted from Preusser, Margraf, and Zlomuzica [15] and slightly modified by adding three additional approach tasks. The BAT for spiders was used to measure fear and avoidance of spiders by requiring participants to approach the spider, located at the very back of the room in a small terrarium, as quickly and as closely as possible, i.e., until their fear became intolerable. The BATs were scored on a scale from 0 (=refused to enter the room) to 13 (=transferred spider into a jar) based on approach distance.

### BAT for heights

The BAT for heights was adapted from Raeder and colleagues [33] and slightly modified by adding one additional approach task. The BAT for heights was employed to measure fear and avoidance of heights. Participants were instructed to climb up a nearby church tower until their fear became intolerable. The BAT was scored in terms of pre-determined stations that gradually increased in height and difficulty. The scoring scale ranged from 0 (=refused to enter the church tower) to 13 (=top of the church-tower). Participants began by looking up at the base of the church tower and were instructed to look down through multiple windows in the church tower as they continued with the BAT. Towards the latter part of the task, participants ascended a spiral staircase that provided them with a direct view into the lower levels of the church tower.

### Subjective fear

During the BATs and exposure treatment, participants reported their subjective fear by using the Subjective Unit of Distress Scale (SUDS [34]); with scores ranging from 0 (=no fear) to 100 (=excessive fear).

#### Exposure Treatment

The exposure treatment was based on the protocol of Öst [35] in a slightly modified form. Each step was first modeled by the experimenter before the participant performed the step herself/himself. Exposure was based on a hierarchy of 14 steps, which increased in difficulty involving 1. watching the spider in a glass at a distance of 150 cm, 2. watching it at a distance of 30 cm, 3. placing one hand on the jar close to the spider, 4. placing both hands on the jar 30 cm away from the face, 5. looking at the spider in the opened jar, 6. watching it crawl in a plastic container, 7. touching it with a pencil, 8. touching it with a thick glove, 9. letting it walk over the thick glove, 10. touching it with a latex glove, 11. letting it walk over the latex glove, 12. touching it with a fingertip, 13. letting it walk over the hand with the arm covered, and finally 14. letting it walk on the bare skin of the hand. All steps were accomplished with the same spider used for the BATs. Fear levels (measured with SUDS) were rated at the beginning of each step and measured continuously. Each step was repeated until a SUDS score of 30 or less was reached before proceeding to the next step. Exposures were considered complete when all steps had been successfully performed or a duration of 120 min had been reached, whichever was fulfilled first.

## Study design and experimental procedures

### Screening

A telephone screening was performed to identify eligible participants. The screening involved an initial assessment of the fear of spiders and heights as well as questions addressing the predefined inclusion and exclusion criteria. Participants who fulfilled the screening criteria were invited to participate in the study.

**Fig. 1:**
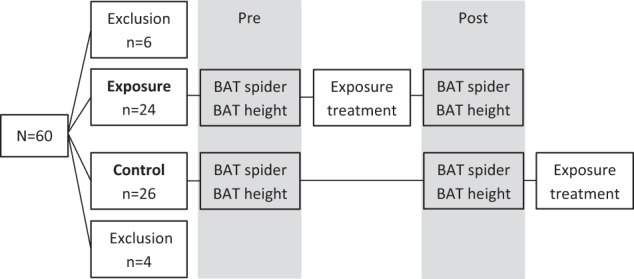
Brief outline of the experimental design. Participants were assigned to the exposure group or control group. Exposure-induced changes in fear and avoidance of spiders were assessed with behavioral approach tests (BATs) prior to (Pre) and after exposure (Post). BATs for heights as the untreated stimulus were conducted to assess exposure treatment generalization. The order of BATs with spiders and heights was counterbalanced across participants. The BAT for spiders was conducted in the treatment room, while the BAT for heights was conducted in a church tower.

Participation in the study consisted of three appointments. The exposure group underwent pre-assessment, exposure treatment, and post-assessment. In contrast, the order for participants in the control group differed in that they first underwent pre- and post-assessment one week apart and then received exposure training. This design allowed to evaluate whether “BAT dosage” (one vs. two BATs) might have had a significant effect on participants’ performance during exposure treatment. Figure 1 provides an outline of the experimental design.

### Pre-assessment

Participants first completed the Mini-DIPS, the demographic questionnaire, the BDI-II, the STAI, the GSE, the spider fear-related questionnaires (i.e., FSQ, SPQ, SBQ), and the AQ. Participants were then given instructions on the BAT and how to use the SUD scale. Thereafter, subjects completed the BAT for spiders and heights, with the order of the BATs being counterbalanced between participants.

### Exposure treatment

One week following the pre-assessment, the exposure group accomplished the exposure treatment.

### Post-assessment

One day following the exposure treatment, the exposure group completed the post-assessment. After a rehearsal of the BAT instructions, the exposure group again underwent the two BATs. During the post-assessment BATs, participants rated their subjective fear level at the same approach distance they had accomplished at the pre-assessment (=defined as the initial approach distance). In the case that their fear at that distance was not yet intolerable, the participants continued with the BATs and reported their level of subjective fear at the closest tolerable distance (=defined as the final approach distance). The difference between initial approach distance (maximal approach achieved during pre-treatment) and final approach distance (maximal approach achieved during the post-treatment) reflects a change in avoidance behavior induced by the exposure treatment for the treated stimuli or the generalization effect for the untreated stimuli.

At the end of this session, participants again completed the spider fear-related questionnaires (FSQ, SPQ, SBQ) and the AQ, and were fully debriefed.

#### Statistical analyses

Data were analyzed using SPSS Version 27 (Armonk, NY, USA). The BATs readouts, approach distance (behavioral level) and fear ratings at the final and initial approach distances (subjective level) were analyzed separately for treatment effectiveness (spider) and exposure treatment generalization (height). Mixed ANOVAs or MANOVAs were performed for statistical analyses. Group (exposure group vs. control group) was used as between-subject factors and time (pre- vs. post-assessment) as within-subjects factor. A result was considered significant at an alpha level of *P* < 0.05.

## Results

### Pre-assessment

The majority of the total sample (68%) indicated that fear of spiders elicited stronger emotional reactions for them than fear of heights. There was no difference in the proportions between the groups (*P* = 0.680). As depicted in Table 1, the exposure group did not differ from the control group in any demographic variables considered, including age, gender, and their questionnaire scores. Most importantly, during pre-assessment, the groups were also comparable regarding their subjective fear levels on the BATs with spiders and heights and their scores on the spider and height fear-related questionnaires (cf. Table 2).

Mean values of primary and secondary outcomes for treated and untreated stimuli are provided in Table 2.

### Exposure therapy effects for treated stimuli (spiders)

#### Primary outcome measures

##### Behavioral scores in the spider fear BATs

**Fig. 2:**
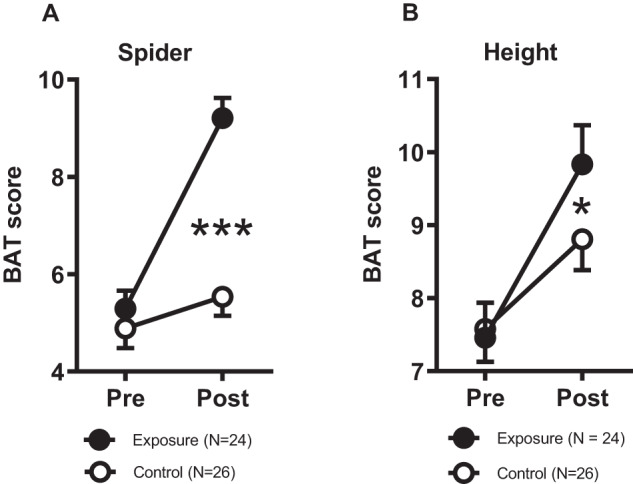
Approach distance in the BAT. **A** Treatment effectiveness measured with the spider BAT; **B** Generalization measured with the height BAT. Squares represent mean ± SEM. Significant time × group interaction effects, with ***P < 0.001, *P < 0.05.

The analysis of the behavioral scores on the spider BAT yielded significant main effects for time (*F*_1,48_ = 82.480, *P* < 0.001) and group (*F*_1,48_ = 16.585, *P* > 0.001) as well as a significant time x group interaction (*F*_1,48_ = 42.034, *P* < 0.001; Fig. 2A). Within-group post-hoc comparisons revealed that both the exposure group (*T*(23) = 7.896, *P* < 0.001) and the control group (*T*(25) = 4.183, *P* < 0.001) showed a significant decrease in avoidance behavior from pre- to post BAT assessments.

##### Fear levels during the spider BATs

**Fig. 3:**
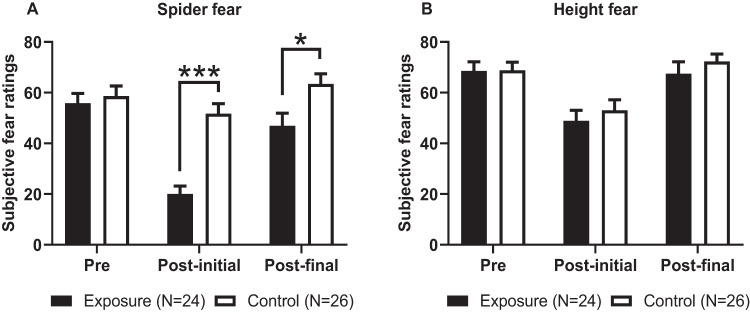
BAT fear level ratings for pre- and post-treatment assessments. **A** Fear ratings during the spider BAT; **B** Fear ratings during the heights BAT. Post-treatment BAT fear ratings have been decomposed into initial and final approach distance. Squares represent mean ± SEM. Significant between-group differences from independent T-tests, with ***P < 0.001, *P < 0.05.

Change in subjective fear ratings at either the initial or final approach distance were analyzed separately, using mixed ANOVAs with time as within-subjects factor and group as between-subjects factor. The analysis of subjective fear ratings at the initial approach distance yielded significant main effects of time (initial: *F*_1,48_ = 61.395, *P* < 0.001), and group (initial: *F*_1,48_ = 14.453, *P* < 0.001). Furthermore, a significant interaction effect was found (initial: *F*_1,48_ = 28.069, *P* < 0.001). Within-group post-hoc comparisons revealed that the exposure group displayed a significant decrease in their subjective fear at the initial approach distance (Pre- vs. Post-initial: *T*(23) = 8.983, *P* < 0.001; T-test for dependent samples) whereas the control group did not (Pre- vs. Post-initial: *T*(25) = 1.855, *P* = 0.075; T-test for dependent samples). The analyses of subjective fear ratings at the final approach distance revealed a significant interaction effect of time and group (final: *F*_1,48_ = 4.510, *P* = 0.039), but no significant main effects of time (*F*_1,48_ = 3.09, *P* = 0.581) or group (*F*_1,48_ = 3.766, *P* = 0.058). Within-group post hoc comparisons revealed that neither the exposure group (pre-vs. post final: T(23) = 1.509, *P* = 0.145; T-test for dependent samples) nor the control group displayed a significant decrease in subjective spider fear at the final approach distance (Pre- vs. post-final: T(25) = 1.620, *P* = 0.118; T-test for dependent samples). Results of fear levels at initial and final approach distance during the spider BAT are displayed in Fig. 3A.

#### Secondary outcome measures

##### Spider fear-related questionnaires.

The MANOVA on the FSQ, SPQ and SBQ yielded significant main effects for time (*F*_1,42_ = 66.435, *P* < 0.001) and group, (*F*_1,42_ = 24.488, *P* < 0.001). Furthermore, a significant time x group interaction was found (*F*_1,42_ = 72.208, *P* < 0.001). As can be seen in Table 2, a decrease of scores over time was only observed in the exposure group, whose scores were significantly lower than those of the control group at post-assessment (*P* < 0.001). Within-group post-hoc comparisons revealed significant reductions between pre- and post-measurements in the exposure (FSQ: *T*(22) = 8.755, *P* < 0.001; SBQ: *T*(23) = 6.865, *P* < 0.001; SPQ: *T*(20) = 3.703, *P* < 0.001; T-test for dependent samples; Table 2), but not in the control group (FSQ: *T*(25) = 0.410, *P* = 0.685; SBQ: *T*(23) = 0.540, *P* = 0.595; SPQ: *T*(25) = 0.178, *P* = 0.860; T-test for dependent samples; Table 2).

These results suggest that exposure treatment indeed leads to the reduction of spider-related fear and beliefs and that this reduction is not the consequence of either test repetition (e.g., regression to the mean effect) or the simple passage of time, since spider-related fear and beliefs measured by the spider-related questionnaires remained stable across the two measurements in the control group.

### Exposure therapy effects for untreated stimuli (heights)

#### Primary outcome measures

##### Behavioral scores in the height fear BATs

Similar to the spider BAT results, there was a significant increase in the height fear BAT scores from the first to the second assessment (main effect for time, *F*_1,48_ = 49.219, *P* < 0.001). No significant main effect of group was found. However, a significant time x group interaction was present (*F*_1,48_ = 4.956, *P* = 0.031; Fig. 2B). Within-group post-hoc comparisons revealed that both the exposure group (*T*(23) = 6.253, *P* < 0.001; T-test for dependent samples) as well as the control group (*T*(25) = 3.539, *P* = 0.02; T-test for dependent samples) displayed a significant increase in approach behavior from the first to the second assessment.

##### Fear levels during the height BAT

Change in subjective fear levels at either the initial or final approach distance were analyzed separately with mixed ANOVAs with time as within-subjects factor and group as between-subjects factor. Due to missing data of *n* = 1 participant in the control group, analysis of fear levels at the initial approach distance was performed with *N* = 49 participants. Data was missing because the participant was not able to re-attain their pre-assessment BAT approach distance at the post-assessment BAT (i.e., showed a closer proximity to the spider at pre- compared to post- assessment). Subjective fear ratings were significantly different between pre- and post- assessments at the initial approach distance (*F*_1,47_ = 33.694, *P* < 0.001, Fig. 3B) but not at the final approach distance (*F*_1,48_ = 0.187, *P* = 0.667). No significant effects of group or time x group interactions were found for the pre- and post-assessments at the initial or final approach distances (All P’s >0.05). Within-group comparisons revealed that both the exposure and control groups displayed a significant decrease in subjective fear ratings from the pre- to the post-assessment at the initial approach distance (Exposure group: *T*(23) = 5.198, *P* < 0.001; control group: *T*(24) = 3.321, *P* = 0.003; T-test for dependent samples), but not at the final approach distance (Exposure group: *T*(23) = 0.203, *P* = 0.841; control group: *T*(25) = 1.470, *P* = 0.154; T-test for dependent samples).

#### Secondary outcome measures

##### *AQ*

**Fig. 4:**
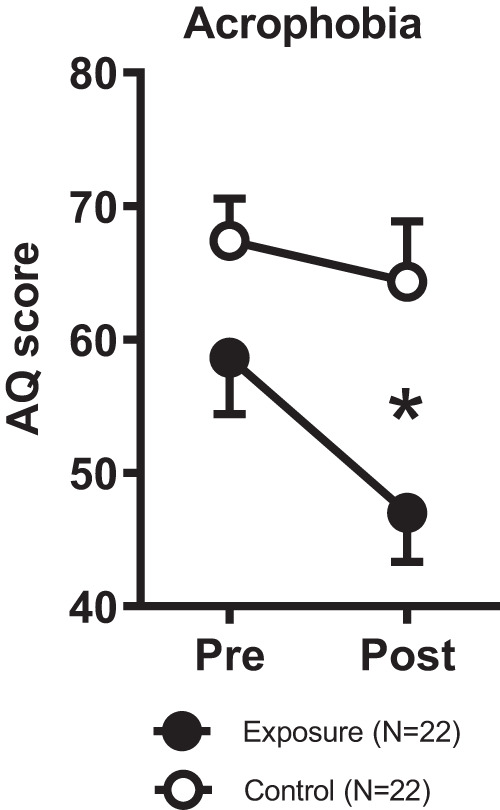
Acrophobia levels in the treatment and control groups. Squares represent mean ± SEM. Significant between-group differences from independent T-tests, with *P < 0.05.

The analysis of the AQ data revealed a significant main effect for time (*F*_1,42_ = 12.918, *P* < 0.001), and group (*F*_1,42_ = 6.379, *P* = 0.015), as well as a significant time x group interaction (*F*_1,42_ = 4.349, *P* = 0.043; Fig. 4). Within group post-hoc comparisons revealed that the exposure group showed a significant decrease in AQ scores from pre- to post measurements (*T*(21) = 3.721, *P* < 0.001; T-test for dependent samples), whereas no such significant decrease was evident in the control group (*T*(21) = 1.168, *P* = 0.256; T-test for dependent samples).

## Discussion

The present study aimed to explore whether exposure-based generalization occurs across fear-eliciting stimuli which belong to different categories and have no perceptual feature overlap. For this purpose, participants with fear of spiders (animal related fears) and heights (natural environment related fears) underwent an exposure therapy targeting their spider fear, while leaving their fear of heights untreated. As expected, participants who underwent exposure therapy showed significant reductions in spider fear from pre- to post-assessment. Remarkably, reductions in fear response were also observed for the untreated fear (i.e., height fear). Generalization of exposure effects to untreated stimuli was indicated by reductions in avoidance behavior (behavioral approach test; BAT) and changes in self-reported height fear symptoms (acrophobia questionnaire; AQ), but not by significant changes in subjective self-assessments of the fear level experienced (subjective units of distress; SUDS). In contrast, the control group did not demonstrate the same level of improvement, neither with spiders nor with heights.

The apparent contradiction between the effects on avoidance behavior and the lack of effect on subjective fear can be explained by considering the temporal dynamics of the beneficial effects of behavioral exposure therapy. It is well-known that behavioral responses often show poor correlation with subjective states [36, 37]. Following from this, initial changes in behavioral responses may precede changes in subjective fear. The latter is likely to be the consequence to mastery and emotion regulation experiences that have been made possible through the reduction in avoidance behavior [38].

Our findings extend previous findings on generalization of exposure therapy effects across stimuli belonging to the same fear category (animal related fears) sharing similar perceptual features [2, 15]. We here provide evidence that exposure-related generalization effects can also be demonstrated across treated and untreated stimuli which do not share perceptual similarities and moreover belong to different fear categories. Exposure, thus, might entail beneficial effects which go beyond the observed reductions in fear and avoidance related to the treatment stimuli. Our findings indicate that exposure effects might lead to an attenuation of fear and avoidance towards fear-eliciting stimuli in general.

While perceptual similarity between these stimuli does not seem to be a prerequisite of such generalization, spiders and height still share some important features in that both are considered to engage biologically prepared defense and aversion mechanisms with a strong evolutionary based component/function [39]. As such, fear-relevant reactions are more easily developed for “prepared” stimuli, i.e., biologically relevant stimuli, such as spiders and heights) than for unprepared stimuli (e.g. flowers and envelopes) [40, 41]. This might explain why fear of spiders and heights are more common in the general population than fear related to unprepared stimuli. Supporting this view, several experiments from fear conditioning studies have demonstrated that fear responses to prepared fear can be more easily acquired [42].

Here, we showed that exposure might counteract such an evolutionary-driven effect in the development and generalization of fear. Exposure effects thus, do not only lead to a reduction in heightened fear responses for a certain subtype of fear-eliciting stimuli, but instead contribute to a downregulation of exaggerated, evolutionary-driven fear responses in general [43].

It is also possible that the observed generalization effect might be the consequence of generalized approach learning, a process analogous to generalized avoidance learning [44]. Just as generalized avoidance learning extends avoidance behaviors beyond stimuli directly associated with negative outcomes, generalized approach learning may involve extending approach behaviors beyond stimuli directly associated with positive outcomes. Furthermore, it should be considered that the present results might also be the consequence of improved self-efficacy due to mastery experiences during exposure or the learning and application of coping-skills in a different context [33, 45].

The generalization of exposure effects on dysfunctional beliefs regarding heights is also interesting. Exposure has been shown to change threat-related beliefs related to certain anxiety disorders and phobia subtypes [46, 47]. Interestingly, patients with spider and height phobia tend to have similar threat-related beliefs [19] while certain beliefs are more tied to the phobia-subtype. Our results are in line with this idea suggesting that a change in threat-related beliefs after exposure to spiders might also be relevant to threat-related beliefs related to heights. However, this explanation is purely theoretical and requires further research.

One much noticed attempt to explain the effectiveness of exposure therapy is based on the inhibitory learning model [13]. According to this model, exposure is based on fear extinction learning, wherein newly formed inhibitory associations compete with and eventually inhibit the retrieval of the original fear memory. Following from this, fear extinction may represent a translational model in the explanation and prediction of post-exposure symptom improvement [48–50]. As such, there is evidence for the generalization of fear extinction [51]. Usually, such extinction generalization has been demonstrated for perceptually related conditioned stimuli, however generalization of fear extinction across conceptual stimuli and stimuli belonging to different categories might also be possible [3, 52]. Thus, generalization of fear extinction towards stimuli belonging to the same category (e.g., biologically relevant stimuli) might account for the herein observed effect, however further studies are needed in order to prove this assumption.

To further our understanding, future research should replicate these findings in diverse anxiety populations. Ideally, these studies should incorporate psychophysiological measures in addition to subjective and behavioral measures and investigate the specific intervention components (e.g. exposure protocols with or without psychoeducation) responsible for the observed effects in more detail. The absence of a long-term follow-up assessment in our study limits our understanding of the long-lasting effects of exposure-based generalization. Including such follow-ups in future research would provide valuable insights. Additionally, testing generalization effects bidirectionally and exploring analogous findings in fear of heights treatment would strengthen the generalization hypothesis and contribute to optimizing the outcomes of exposure therapy.

Since many fears and phobias have their onset in childhood and have been shown to be strongly predictive of other anxiety disorders [53], applying exposure as early as in childhood could have the potential to reduce the risk of onset of other disorders and reduce treatments of phobias in both length and expense [54]. Future studies using a similar design to our study in children with specific fears would therefore be of great value.

Another clinically important implication of our study is related to the fact, that exposure has been commonly reported to be an unpleasant experience. The vast majority of individuals suffering from phobias do not seek treatment as they are too afraid of having to confront their fears [55]. Moreover, as phobic individuals often suffer from multiple phobias, designing exposure therapy in a way that requires confrontation with one fear only rather than multiple exposure sessions for each existent fear, individuals’ distress level and threshold for entering therapy could be significantly reduced.

In conclusion, our findings provide first evidence for generalization of exposure effects in spider phobia to another perceptually unrelated fear-evoking stimulus category, i.e., height. Studies investigating how such generalization effects can be further promoted are therefore warranted.

## Data Availability

The data that support the findings of this study are available from the corresponding author, AZ, upon reasonable request.
